# Design of Drug Delivery Systems Containing Artemisinin and Its Derivatives

**DOI:** 10.3390/molecules22020323

**Published:** 2017-02-20

**Authors:** Blessing Atim Aderibigbe

**Affiliations:** Department of Chemistry, University of Fort Hare, Alice Campus, Eastern Cape 5700, South Africa; blessingaderibigbe@gmail.com

**Keywords:** artemisinin, artesunate, artemether, arteether, drug delivery systems

## Abstract

Artemisinin and its derivatives have been reported to be experimentally effective for the treatment of highly aggressive cancers without developing drug resistance, they are useful for the treatment of malaria, other protozoal infections and they exhibit antiviral activity. However, they are limited pharmacologically by their poor bioavailability, short half-life in vivo, poor water solubility and long term usage results in toxicity. They are also expensive for the treatment of malaria when compared to other antimalarials. In order to enhance their therapeutic efficacy, they are incorporated onto different drug delivery systems, thus yielding improved biological outcomes. This review article is focused on the currently synthesized derivatives of artemisinin and different delivery systems used for the incorporation of artemisinin and its derivatives.

## 1. Introduction

Artemisinin is obtained from the Chinese medicinal herb *Artemisia annua* [1]. Its structure is a sesquiterpene lactone containing an internal peroxide bridge which makes it different from other available drugs [1]. Some of its derivatives such as artesunate, artemether, artenimol, artelinic acid and arteether are referred to as semisynthetic derivatives [1]. The therapeutic applications of artemisinin and its derivatives is wide and huge. They are used to reduce angiogenesis; they exhibit antitumor and anticancer activities [2]; they are used for the treatment of joint pain; epilepsy; liver problems; menstrual pain and loss of appetite [3]. They also exhibit anti-inflammatory activity, sedative activity, treat insomnia and gastrointestinal disease, exhibit antibacterial activity and are useful for the treatment of ulcers, skin diseases and wounds [3]. They build-up the healthy cells, prevent infections, destroy existing infections and inhibit viral infections [3,4]. They are effective for the treatment of malaria and protozoan infections [5,6].

Despite the effectiveness of artemisinin in the treatment of several diseases, it is limited in the treatment of malaria by its cost when compared to other antimalarials [7], poor bioavailability and solubility, short half-life, toxicity and drug resistance [8]. Due to the aforementioned pharmacological limitations, several researchers are designing derivatives with enhanced therapeutic effects and some researchers have developed delivery systems in which artemisinin and its derivatives are incorporated to produce improved therapeutic effects. This review will focus on the recent trends in the incorporation of artemisinin and its derivatives onto delivery systems and the biological evaluations of these systems.

## 2. Artemisinin Derivatives

**Figure 1:**
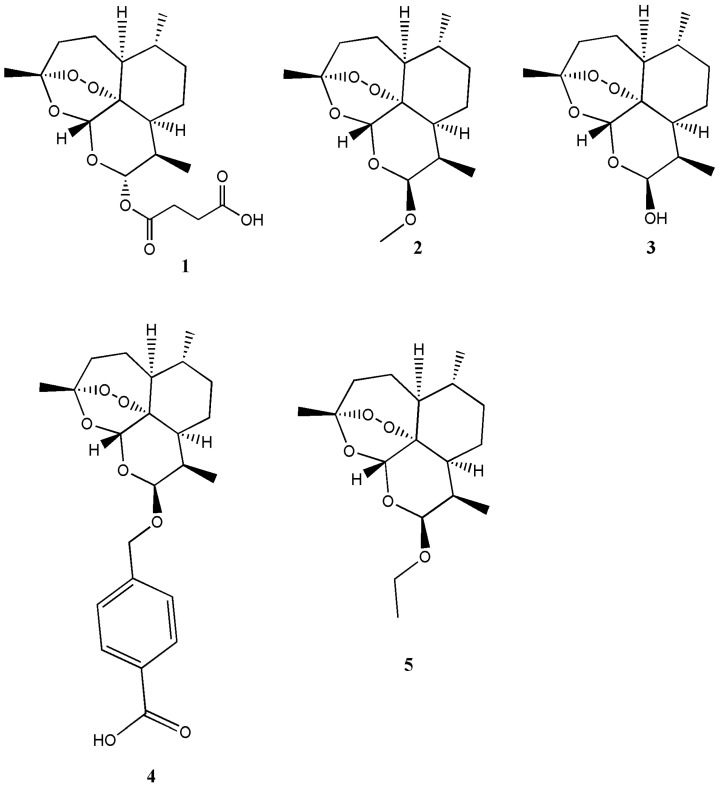
Semisynthetic derivatives of artemisinin: artesunate (**1**), artemether (**2**) Dihydroartemisinin (**3**), artelinic acid (**4**) and arteether (**5**).

**Figure 2:**
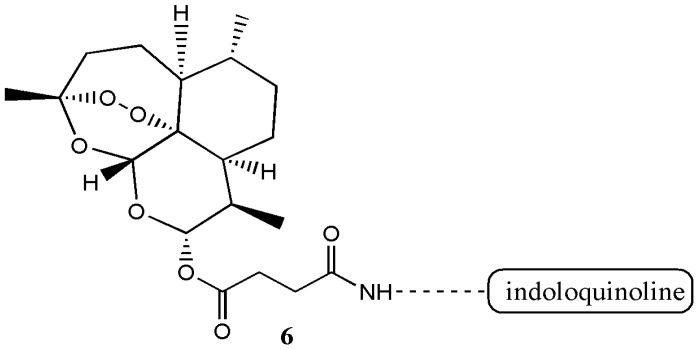
Artesunate-indoloquinoline hybrid **6**.

Presently, there are several known derivatives of artemisinin such as artesunate (**1**), artemether (**2**), artenimol (**3**), artelinic acid (**4**) and arteether (**5**) (Figure 1) [1]. There are also several derivatives of artesiminin derivatives/hybrid compounds containing artemisinin that have been reported with good biological activity in vivo/in vitro when compared to the free artemisinin (Table 1). Wang et al. prepared the artesunate-indoloquinoline hybrid **6** that was active as an antimalarial [9] (Figure 2).

**Figure 3:**
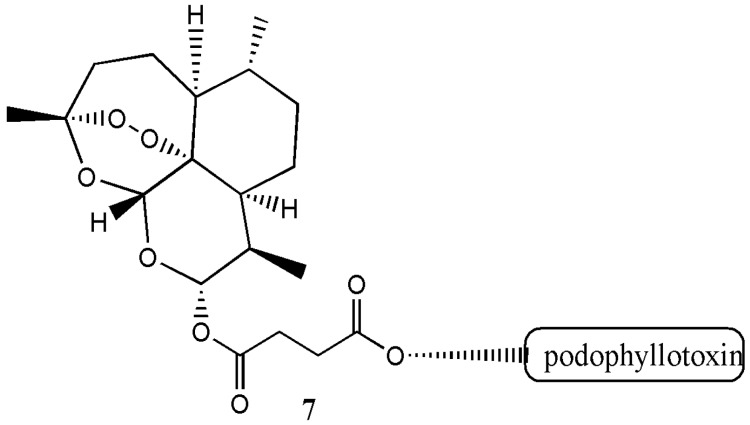
Artesunate-podophyllotoxin analogue **7**.

In vitro evaluation of the cytotoxicity of the hybrid compound on normal cells revealed a decreased cytotoxicity. The antimalarial activity of the hybrid compound was good and it acted as an excellent β-haematin inhibitors when compared to the free drugs [9]. Zhang et al. prepared artesunate-podophyllotoxin analogue **7** with good anticancer activity in vitro [10] (Figure 3).

**Figure 4:**
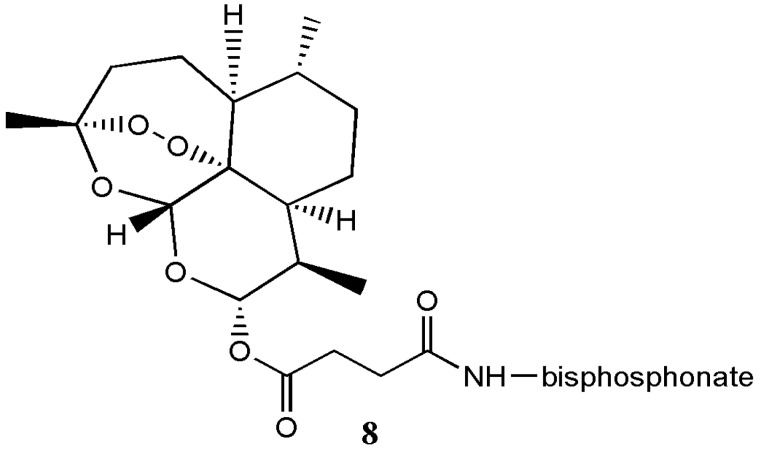
Artesunate α-aminophosphonate analogue **8**.

He et al. synthesized artesunate α-aminophosphonate analogue **8** with good antimicrobial activity that was further enhanced when combined with roxithromycin, an antibiotic (Figure 4) [11].

**Figure 5:**
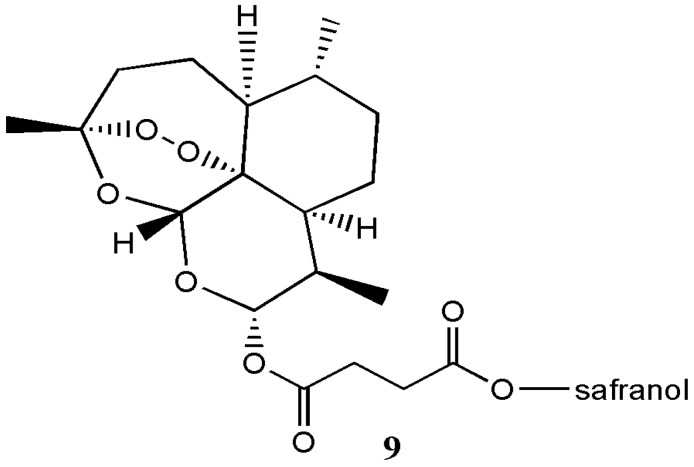
Artesunate-safranol analogue **9**.

Griesbeck et al. prepared artesunate-safranol analogue **9** by DCC coupling reaction of safranol and artesunic acid, followed by photo-oxygenation (Figure 5) [12]. However, no biological evaluation was performed on the hybrid compound.

**Figure 6:**
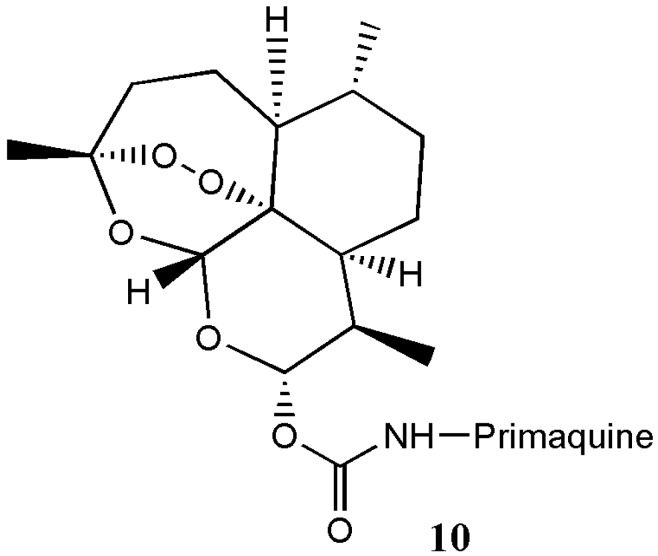
Primaquine-artemisinin hybrid **10**.

Capela et al. prepared primaquine-artemisinin analogue **10** with effective antimalarial activity against drug resistant *P. falciparium* (Figure 6) [13].

**Figure 7:**
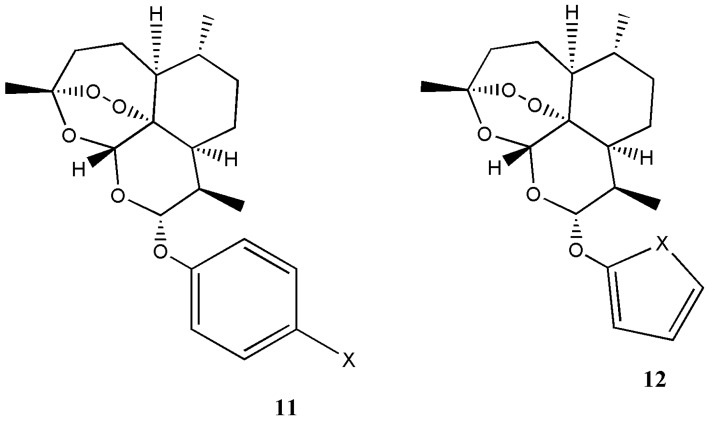
Artemisinin derivatives **11** and **12**.

Ying et al. prepared arteminisin derivatives containing Mannich base group and artesunate with excellent antimalarial activity and good stability [14]. Chand et al. prepared β-ether derivatives of dihydroartemisinin in excellent yield and high diastereoselectivity [15]. Opsenica et al. reported synthesis of artemisinin derivatives **11** and **12** with enhanced antimalarial activity (Figure 7) [16].

**Figure 8:**
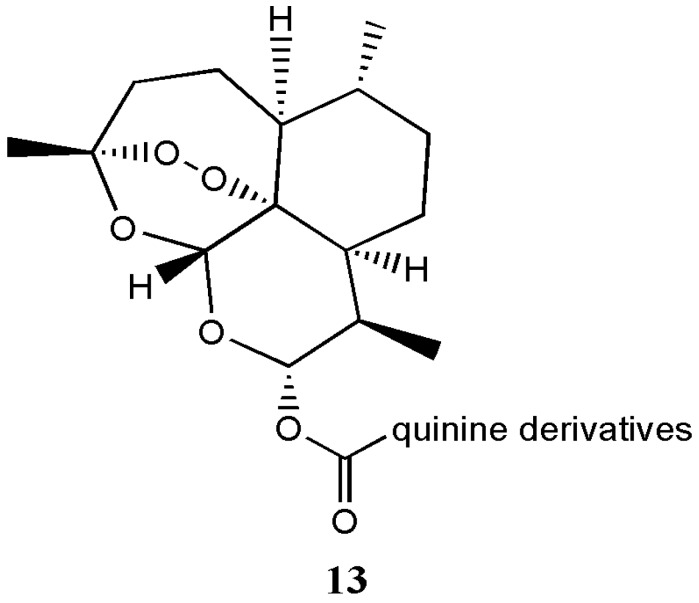
Artemisinin-quinine hybrid **13**.

Walsh prepared artemisinin-quinine hybrids **13** with good antimalarial activity (Figure 8) [17]. In vitro evaluation of the compound on 3D7 and FcB1 strains of *Plasmodium falciparum* indicated that the potent activity of the hybrid compound was higher than that of the free artemisinin drug [17]. Joubert et al. prepared artemisinin-acridine hybrids effective against malaria and cervical cancer in vitro [18]. Pandey et al. prepared a pyrrolidine-acridine-artemisinin hybrid with antimalarial activity suitable for combination therapy [19].

**Figure 9:**
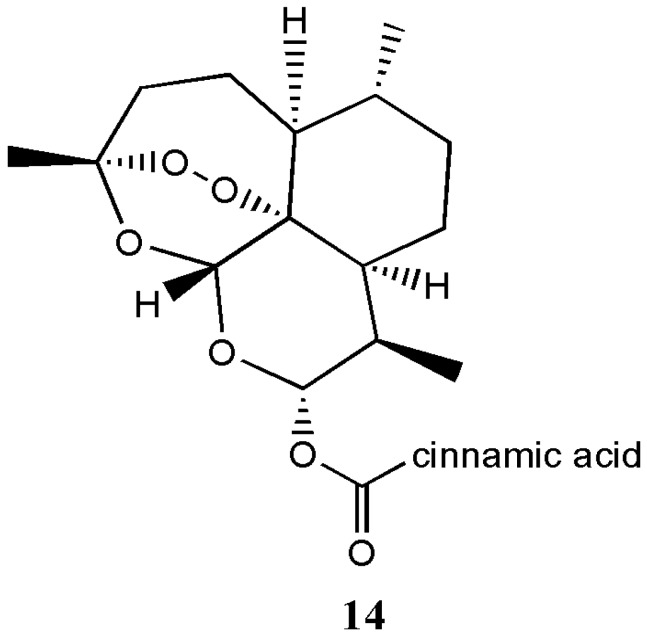
Dihydroartemisinin-cinnamic acid hybrid **14**.

Guo et al. prepared artemisone and artemiside derivatives from dihydroartemisinin for treatment of severe murine malaria in vivo [20]. Wei et al. prepared artemisone derivatives from dihydroartemisinin with antitumor activity [21,22]. Soomro et al. prepared artemisinin derivatives effective for the treatment of cancer and infections [23]. Xu et al. prepared dihydroartemisinin-cinnamic acid hybrids **14** that were active against selected cancer cell lines (Figure 9) [24].

## 3. Pharmacokinetics of Artemisinin and Its Derivatives

Derivatives of artemisinin can be administered orally, intravenously, intramuscularly and rectally [25,26]. They have been reported to exhibit neurotoxicity which is dependent on the nature of the formulation and mode of administration [1]. In the antimalarial activity of artemisinin and its derivatives, the principal bioactive metabolite is dihydroartemisinin and the activity is considered to be due to the presence of the endoperoxide bond [27]. Artemisinin free radical is also believed to form a covalent bond with the heme or parasite proteins thereby inhibiting the production of hemozoin [27,28]. Artemisinin half-live ranges between 2 to 5 h, whereas the half-lives or artemether and artesunate range between 2 to 4 h and less than 1 h, respectively [1]. The metabolism of artemisinin is primarily mediated by the genes CYP2B6 and CYP3A4. Artesunate is metabolised by CYP2A6 to dihydroartemisinin, an active metabolite which is responsible for its antimalarial activity. Artemether is also rapidly metabolised to dihydroartemisinin by CYP3A4 and CYP3A5 [1]. Arteether is metabolised by CYP3A4 to dihydroartemisinin. The active metabolite, dihydroartemisinin is converted to inactive metabolites by glucuronidation. Dihydroartemisinin is eliminated in the bile as minor glucuronides [1].

The presence of the endoperoxide moiety is also responsible for the anticancer activity of artemisinin and its derivatives [29,30]. The absence of the endoperoxide moiety reduces significantly their cytotoxic effects. Iron and heme metabolism is important in the anticancer activity of artemisinin because it enhances its cytotoxicity [29,31,32,33]. It has been postulated that iron-activated artemisinin produces alkylating carbon-centered radicals and radical oxygen species that results in DNA damage, enhanced apoptosis, growth arrest and reduced angiogenesis [29].

Artemisinin derivatives such as artesunate has been reported to exhibit antiviral activity. It hinders in vitro replication of human cytomegalovirus (HCMV) and β-herpes virus that are responsible for lifelong infections in humans. The mode of action was postulated that it inhibit central regulatory processes of HCMV-infected cells thereby interfering with host-cell type and metabolism requirements for HCMV replication [2,34,35].

## 4. Delivery Systems Loaded with Artemisinin and Its Derivatives

### 4.1. Polymer-Drug Conjugates

**Figure 10:**
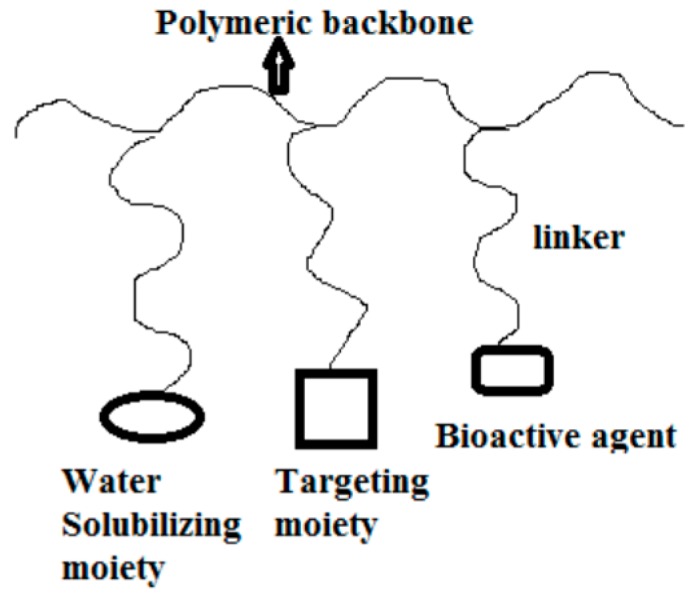
A schematic diagram of polymer-drug conjugate.

Polymer-drug conjugates are polymeric therapeutics which consist of a biodegradable polymeric backbone, a targeting moiety, a bioactive molecule with low molecular weight incorporated to the polymer via covalent bond and a bio responsive linker (Figure 10) [36].

Incorporation of bioactive agents into polymers enhances their solubility and pharmacokinetic profiles, increases plasma half-life, reduces rate of clearance by the kidneys or liver and protects the bioactive agents against premature degradation [36,37,38,39] (Table 2). Two or more bioactive agents can be incorporated onto polymeric carriers for combination therapy.

Liu et al. prepared polymer-drug conjugates for targeted delivery to the tumor from transferrin-eight-arm-polyethylene glycol nanoparticles loaded with dihydroartemisinin [40]. The role of targeting ligand in polymer-drug conjugates was demonstrated using transferrin as a targeting moiety. The rate of proliferation of cancer cells is usually rapid with their high demand for iron than the normal cells, resulting in an increased expression of transferrin on the surface of cancer cells. In vitro cytotoxicity evaluation on Lewis lung carcinoma cells revealed that incorporating dihydroartemisinin into the polymer resulted in enhanced solubility and long circulating half-life of the incorporated drug. In vivo evaluation further revealed that the conjugates inhibited tumor growth in mice bearing Lewis lung carcinoma than the free drug [40].

Incorporation of dihydroartemisinin onto the polymer resulted in enhanced water solubility, improved their stability and enhanced bioavailability. The presence of the targeting moiety, transferrin resulted in enhanced cellular uptake. No side effects were observed in the polymer-drug conjugates when compared to the free dihydroartemisinin suggesting that the poor water solubility of artemisinin compounds is responsible for their toxic side effects.

Wang et al. incorporated dihydroartemisinin to hydroxypropyl-β-cyclodextrin for oral administration. Incorporating dihydroartemisinin to the modified cyclodextran resulted in enhanced solubility and stability of dihydroartemisinin [41]. However, it is important to mention that high dose of cyclodextrin can result in toxicity. In order to overcome toxicity, auxiliary substances are employed. Wang et al. used different auxiliary substances resulting in a reduced uptake of cyclodextrin and enhanced biological activity of the formulation.

Dal et al. incorporated dihydroartemisinin onto polyethylene glycol carriers in order to overcome their poor water solubility and enhance their bioavailability of dihydroartemisinin. The conjugates were soluble in water, inhibited tumor growth and enhanced blood circulation half-time significantly in vivo [42]. The release of the incorporated drug from the conjugates was slow in vitro because of the hydrolysis of the ester bond between the polymer and the drug, indicating that the type of drug linker influences drug release mechanisms. Yaméogo et al. incorporated artemisinin onto cyclodextrin via vinyl-acyl fatty esters. PEGylated amphiphiles were incorporated onto the cyclodextrin nanostructures. In vitro antimalarial evaluation showed that the suspensions inhibited the growth of cultured *Plasmodium falciparum* [43]. Burst effects was observed and the stability of the formulation was dependent on the high drug content. The sustained release of the drug from the formulation was influenced by the interaction of the artemisinin with cyclodextrin. Xiao et al. prepared *C*-10-phenoxy artemisinin-chitosan conjugate. Artemisinin derivative was covalently bonded to chitosan resulting in enhanced solubility of artemisinin than the free artemisinin drug [44]. The percentage incorporation of artemisinin onto chitosan was dependent on the ratio of chitosan to artemisinin. Kumar et al. prepared poly(organophosphazenes) carriers loaded with primaquine and dihydroartemisinin [45]. The degradation of the conjugates was higher at acidic pH. The nature and position of functionalities on the conjugates influenced the degradation behaviour of the conjugates. In vivo evaluation on *P. berghei* infected mice further revealed that the formulation is a potential therapeutic useful in combination therapy for the treatment of resistant malaria [45].

Incorporation of artemisinin derivatives onto polymers resulted in enhanced water solubility [40,41,42,43], improved stability [41], enhanced bioavailability by extending the circulation half-life of the drugs [40,41] and useful for combination therapy [45]. The release of the artemisinin and its derivatives from the conjugates was dependent on the pH whereby the bond between the conjugated drug and the polymer undergo hydrolysis resulting in the release of the drug at the targeted site [40,42,43,45]. The enhanced water solubility, stability and extended blood circulation of the polymer-drug conjugates resulted in their improved anticancer and antimalarial effects when compared to the free drugs that are limited by poor water solubility and poor bioavailability [40,41,42,43,44,45]. Polymer-drug conjugates are potential delivery systems for the delivery of artemisinin and its derivatives with enhanced therapeutic efficacy. However, polymer-drug conjugates containing artemisinin derivatives in vitro drug release was quick in the presence of esterase suggesting that the type of drug linkers employed determines their susceptibility to hydrolysis [40]. The design of the polymer-drug conjugates affect their therapeutic outcomes such as the nature of targeting moieties used affect the specificity of the conjugates [40], materials used affect their degree of toxicity [41], the type of drug linkers determine the drug release mechanism from the conjugates [43] and the position of functionalities on the conjugates affect their rate of degradation [45].

### 4.2. Micelles

**Figure 11:**
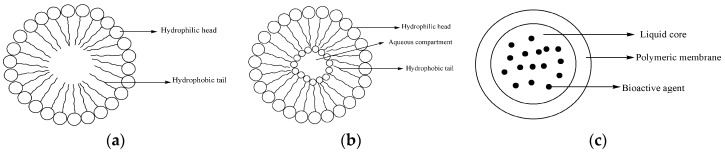
(**a**) Schematic diagram of a micelle; (**b**) Liposomes; (**c**) Nanocapsule loaded with bioactive agent.

Micelles are polymeric delivery systems that are used to deliver several bioactive agents. They are composed of amphiphilic block copolymers forming a hydrophobic core used for encapsulation of lipophilic drugs (Figure 11a) [50]. The size of polymer micelles ranges between 10 to 200 nm [50]. Their structure enhances prolonged circulation in the bloodstream and are useful for sustained drug release mechanisms (Table 2) [50].

Bhadra et al. developed a polymeric amphiphilic micellar system from methoxypolyethylene glycol loaded with artemether [46]. The formulation enhanced drug stability with prolonged release of artemether over a period of two days [46]. However, the system was found to be toxic as a result of its densely clubbed hyper-branched micellar structures and the slow rate of degradation. This finding indicate that the design of the micelles is very important because poor designs can influence their degree of toxicity. Jabbarzadegan et al. encapsulated arteether into polyurethane-based nanomicelles [47]. The formulation exhibited fast release of arteether at pH 5.4 with significant inhibition of the growth of 4T1 cell line [47]. Wang et al. conjugated artemisinin to PEG-PCL micelles for delivery to highly metastatic tumor [48]. The formulation revealed high cellular uptake and inhibition effects on cancer cell lines with good antitumor efficacy [48]. The cellular uptake, selectivity and low toxicity of the formulation was due to the presence of lymphatics-homing peptide incorporated onto the micelles. The design of micelles influence their biological activity and therapeutic efficacy. Lu et al. encapsulated dihydroartemisinin into methoxypoly(ethylene glycol)/poly(l-lactic acid) with anticancer effects. The micelles were worm-like with particle size of 130 nm [49]. In vitro drug release from the micelles was dependent on the stability of micelles, pH, rate of biodegradation of the micelles and rate of diffusion of the incorporated drug from the micelles. Burst drug release effect was observed and the conjugates were selective towards selected cell lines. Their effects on normal cell lines was minimal and potent on cancer cell lines. This indicated that micelles can reduce the toxic side effects associated the incorporated drug [49].

Incorporation of artemisinin and its derivatives to polymeric micelles resulted in improved drug stability, enhanced cellular uptake and controlled release of the drugs resulting in increased bioavailability [46,47,48,49]. In vitro evaluation of the micelles loaded with artemisinin and its derivatives inhibited the growth of cancer cell lines when compared to free artemisinin and its derivatives [47,48,49]. The release of the drugs was pH dependent [46,47,49]. However, the application of micelles for artemisinin is limited by poor drug incorporation and burst effects [49]. It is important to mention that the design of micelles is very important because it influence their degree of toxicity [46], rate of biodegradation [46,47,48,49] and the rate of diffusion of the incorporated drugs from the micelles [47,48,49].

### 4.3. Liposomes

Liposomes contain amphiphilic molecules which are composed of an aqueous core with lipid bilayer that separates the inner aqueous core from the outside (Figure 11b) [51]. They improve the therapeutic effects of encapsulated drugs by enhancing drug absorption, extending biological half-life and reducing drug toxicity [51]. They are biodegradable and biocompatible. They interact with the cells by: simple adsorption, endocytosis, fusion with cell membranes and by lipid exchange [51]. They have been employed for the delivery of artemisinin and its derivatives with good biological activity than the free artemisinin and its derivatives (Table 3).

Liposomes loaded with artemisinin and its derivatives have been reported to be effective as anticancer agents and antimalarials when compared to free artemisinin and its derivatives. Chen et al. reported artemether encapsulated in liposomes with significant sonodynamic anticancer activity [52]. The application of ultrasound in the treatment of cancer is a non-invasive approach and it can penetrate deep tissues making it a potential approach for the treatment of cancer. Liposomes loaded with sonosensitizers and artemether in vitro killing effects on tumor cells was influenced by ultrasound radiation. Ultrasound radiation triggered drug release from liposomes by formation of cavities on the liposomes membranes resulting in rapid drug release and enhanced cellular uptake. Neda et al. prepared nanoliposomal formulation of artemisinin with anticancer activity from poly(ethyleneglycol), phophatidylcholine and cholesterol [53]. The formulations were stable, non-toxic and their biological activity was influenced by the materials used for their preparation. Isacchi et al. prepared artemisinin-based liposomal formulations [54]. In vivo evaluation on *P. berghei* NK-65 infected mice at the dosage of 50 mg/kg/days alone and in combination with curcumin (100 mg/kg/days) revealed that the liposomal formulation containing artemisinin in combination with curcumin cured all malaria infected mice [54]. The curcumin loading efficiency onto the PEGylated liposomes was low which was as a result of the small size of the liposomes. In PEGylated liposomes loaded with artemisinin and curcumin, the synergistic effect of curcumin was not significant which is attributed to a high dosage of artemisinin in the combination therapy [54].

Li et al. developed a liposomal formulation with encapsulated paclitaxel and artemether [55]. In vivo studies in brain glioma bearing rats revealed that the formulation was effective for the treatment of invasive brain glioma [55]. The formulation was able to penetrate blood brain barriers, destroy brain cancer vasculogenic mimicry channels and eliminate brain cancer stem cells thereby overcoming the barrier that is common in drugs used for the treatment of invasive brain glioma. Isacchi et al. also reported artemisinin-loaded polyethylene glycol-based liposomes [56]. The drug encapsulation efficacy was more than 70% in vitro. Investigation on mice showed that the liposomal formulations blood-circulation time was prolonged more than the free drug. The half-life of artemisinin in the liposomal formulation was also improved by more than 5-fold signifying that the formulation is a potential therapeutic for the treatment of tumors and parasitic diseases [56]. The significant effect of encapsulation of aremisinin to liposomes was the extended blood circulation. Gharib et al. loaded artemisinin and transferrin onto magnetic nanoliposomes prepared from lipids [57]. The formulation was thermo-sensitive and exhibited high antiproliferative activity influenced by the presence of a magnetic field [57]. A combination of magnetic nanoliposomes and transferrin resulted in targeted delivery of the formulation and hence, enhanced the therapeutic efficacy. Magnetic liposomes are useful as smart drug delivery systems and magnetic resonance imaging. Jin et al. assessed the anticancer effects of artesunate loaded nanoliposomes [58]. The formulation inhibited tumor growth and the inhibitory effect of nanoliposomes containing artesunate was 32.7%, whereas artesunate API was 20.5%. HepG-2 cells treated with nanoliposomes containing artesunate showed dose-dependent apoptosis. The antitumor effect of nanoliposomes containing artesunate and its effect on human hepatoma HepG2 cells was significant [58]. The inhibitory effects of the drug loaded liposomes was dependent on the concentration of the drug. Dadgar et al. prepared pegylated-based nanoliposomal formulation of artemisinin [59]. The nanoliposomes were prepared from phosphatidylcholine and polyethylene glycol. Incorporation of artemisinin to the liposomes enhanced its cytotoxic effects [59]. However, the percentage encapsulation of artemisinin in the formulation was low. Righeschi et al. prepared liposomal formulations of dihydroartemisinin [60]. The formulations were not toxic, exhibited anticancer activity and can be administered parenterally. However, formulations prepared from polyethylene glycol, exhibited reduced cellular uptake which is attributed to the hydrophilic steric hindrance of polyethylene glycol.

The percentage drug encapsulation of the liposomes was influenced by their size. Liposomal formulations containing artemisinin and its derivatives were effective in the treatment of cancerous and parasitic diseases. The formulations reduced the toxic side effects of the incorporated drug, extended blood circulation time with enhanced inhibitory effects [52,53,55,56,57,58,59,60]. However, liposomes prepared for the encapsulation of artemisinin derivatives were limited by their poor storage stability and low drug loading [59,60]. The type of polymers used to prepare liposomes affected their cellular uptake [60]. The inhibitory effects of liposomes loaded with artemisinin derivatives was dependent on the concentration of the loaded drug [58].

### 4.4. Nanocapsules

Nanocapsules are a class of nanoparticles composed of a core and a protective shell where bioactive agents are encapsulated (Figure 11c) [68]. They have several advantages such as sustained drug release mechanisms, improve drug bioavailability and reduce drug toxicity [68]. Due to the aforementioned advantages, they have been used to encapsulate artemisinin and its derivatives.

Chen et al. encapsulated artemisinin to nanocapsules prepared from chitosan, gelatin, and alginate with controlled drug release mechanism [61]. Nanocapsules encapsulated with artemisinin exhibited prolonged release mechanism (Table 3) [61]. The hydrophilic property of artemisinin was enhanced by encapsulating it onto nanocapsules. Tran et al. encapsulated artesunate onto chitosan-coated lipid nanocapsule. In vitro evaluation on different cancer cell lines showed that the formulation exhibited excellent anticancer activity towards MCF-7 and MDA-MB-231 with enhanced drug stability than the free artesunate [62]. Meng et al. performed esterification reaction between poly(ethylene glycol) monomethyl ether and artesunate resulting in nanocapsules [63]. The release of artesunate from the nanocapsules was controlled by the ester bond. The cytotoxicity effect of formulation on L1210 and MCF7 cell lines was decreased compared to the free drug [63]. Xiao et al. prepared nanocapsules containing artesunate by microcrystallization method with a potential to increase the bioavailability of artesunate [64]. Encapsulation of artemisinin and its derivatives onto nanocapsules resulted in improved drug stability, prolonged drug release mechanism and enhanced bioavailability [61,62,63,64]. The drug linker used influenced the release mechanism of artemisinin from the nanocapsules [63].

### 4.5. Niosomes

Niosomes are non-ionic surfactant vesicles similar to liposomes [69]. They are more stable than liposomes and they have some advantages which make them better than the liposomes. They can be used to incorporate hydrophilic and lipophilic drugs in aqueous layer and vesicular membrane, respectively [69].

Dwivedi et al. designed niosomes for encapsulation of artemisone, a 10-aminoartemisinin derivative with antitumor activity (Table 3) [65]. In vitro evaluation on cancer cell lines, human melanoma A-375 cells and human keratinocytes (HaCaT) revealed that the formulation cytotoxic effects towards the melanoma cells were highly selective. No significant toxicity towards the normal skin cells was observed. The formulation inhibited the growth of the cancer cell lines more than the free drug [65]. Asgharkhani et al. prepared a niosomal formulation containing artesunate [66]. The release of artesunate from the formulations was slow, with no burst effect. The inhibitory effects against MCF-7 and C6 cell lines was significant [66]. Incorporation of artemisinin derivatives onto niosomes enhanced the selective cytotoxicity of the formulation [65] and the cytotoxic effects was also significant when compared to the free artemisinin derivatives [65,66]. The application of niosomes in drug delivery is very new is still at its infancy stage and they are physically unstable.

### 4.6. Ethosomes

Ethosomes are soft lipid vesicles. They are composed of phospholipids, alcohol and water [70]. They are used for transdermal and dermal drug delivery. They are easy to prepare, non-toxic and patient compliance. However, their use is limited by their poor yield [70].

Shen et al. prepared artesunate-loaded ethosomes with antimalarial activity [67]. The formulation enhanced the accumulation permeation of artesunate significantly over a period of 8 h after administration than the free drug. The formulation killed *Plasmodium* parasite significantly with prevention of a resurgence of the infection (Table 3) [67]. The skin penetrating capacity of the ethosomes containing artemisinin derivatives indicate their ability to interact with the microstructure of the skin resulting in enhanced permeability. However, overcoming the barrier of the skin for enhanced transdermal permeation of drugs is challenging. Presently there is only one research report on the design of ethosomes for the delivery of artemisinin derivatives and more studies are required to evaluate the stability of ethosomal systems in long-term storage.

### 4.7. Carbon-Based Materials

Carbon based materials such as graphene, carbon nanotubes and fullerenes have been employed in the preparation of drug delivery systems (Table 4). They have unique properties such as large surface area and can be modified so as to enhance their biocompatibility and biodegradability [71].

Rezaei et al. incorporated artemisinin onto multi-walled carbon nanotubes without altering the properties of the drug [72]. In vitro evaluations on K562 cancer cell lines revealed the inhibition effects on cancer cell growth by the formulation was higher than free artemisinin [72]. Zhang et al. prepared a multi-walled carbon nanotubes-hyaluronic acid formulation loaded with artemisinin [73]. Transferrin was employed as a targeting ligand and the formulation exhibited enhanced antitumor efficacy in tumor-bearing murine model [73]. Zhang et al. grafted hyaluronic acid onto fullerene containing transferrin and artesunate with tumor-targeting efficacy [74]. The formulation exhibited good antitumor efficacy in tumor-bearing murine model [74]. Modification of carbon nanotubes with a targeting moiety resulted in targeted drug delivery [73,74]. Incorporation of artemisinin and derivatives to the modified CNTs enhanced their anticancer activity. However, there is a pressing need to evaluate the possible toxic side effects associated with the use of CNT over an extended period of time.

### 4.8. Nanoparticles

#### 4.8.1. Lipid Based Nanoparticles

Lipid-based nanoparticles can be classified as solids and nanostructured lipid-based nanoparticles. Solid lipid-based nanoparticles size ranges between 50–1000 nm after encapsulation of bioactive agents [75]. They are biocompatible, biodegradable and their preparation is not expensive [75]. They consist of a core and their stability is enhanced by the addition of surfactant coating. However, they are limited by particle agglomeration which usually results in drug burst release effects [75]. They have unique crystal networks making it possible to load bioactive agents in molecular form. Nanostructured lipid-based nanoparticles have amorphous structures and the drug burst effect is reduced [75]. Their particle sizes ranges between 100 to 500 nm and they are useful for the delivery of bioactive agents. Table 4 shows the delivery of arteminisin derivatives with enhanced therapeutic efficacy.

Lipid-based nanoparticles loaded with artemisinin and derivatives have been administered orally with enhanced therapeutic effects. Zhang et al. developed artemisinin dimer piperazine derivatives incorporated onto lipid-based nanoparticles [76]. The formulation exhibited enhanced inhibition of cell-proliferation effects than the free drug [76]. The release of artemisinin was pH sensitive which is associated with the presence of the pH sensitive moiety, piperazine moiety. The rate of release of artemisinin decreased with increase in the environmental pH. Dwivedi et al. prepared solid lipid nanoparticles loaded with areether for the treatment of cerebral malarial [77]. The release of areether was slow and in vivo evaluation showed that the drug loaded lipid enhanced the oral bioavailability of the drug [77]. The formulation was useful and effective when administered orally. Incorporating areether onto solid lipid nanoparticles protected the drug from acidic pH of the stomach thereby improving their bioavailability and therapeutic efficacy. Lipid based nanoparticles loaded with artemisinin derivative have been administered intravenously. Zhang et al. assessed the pharmacokinetics and tissue distribution of nanostructured lipid carrier loaded with dihydroxyartemisinin after intravenous administration [78]. The system showed decreased systemic toxicity with sustained drug release mechanism [78]. In a research report by Aditya et al. artemeter was loaded onto lipid nanoparticles and their therapeutic efficacy after parenteral administration was evaluated [79]. The formulation was not toxic and suitable for parenteral delivery. In vivo evaluation further confirmed that the formulation was biocompatible and useful for the treatment of malaria [79]. The application of lipid-based nanoparticles has several advantages. However, their application is limited by their tendency to gelation and low drug incorporation.

#### 4.8.2. Polymer-Based and Inorganic-Based Nanoparticles

Polymer-based and inorganic materials have been used for the delivery of bioactive agents because they are biodegradable and biocompatible. Some researchers have investigated their applications in the delivery of artemisinin and its derivatives with excellent therapeutic effects (Table 4).

##### 4.8.2.1. Polymer-Based Nanoparticles

Polymeric-based nanoparticles have been employed for the treatment of leishmaniasis in vivo. Want et al. prepared poly(lactic-co-glycolic)acid nanoparticles loaded with artemisinin with antileishmanial efficacy [80]. In vivo evaluation on BALB/c model with visceral leishmaniasis showed a significant reduction in the parasite load in the liver and spleen than the free artemisinin [80]. The artemisinin-loaded nanoparticles refurbish the expression of CD80 molecules resulting in the restoration of appropriate effector T-cell response. Gupta et al. prepared artemisinin HCl nanoparticles by solvent evaporation method from poly(ε-caprolactone) [81]. The in vitro release mechanism of artemisinin from the nanoparticles was sustained over a period of 24 h [81]. In the anticancer effects of polymeric nanoparticles loaded with artemisinin derivative, Nguyen et al. loaded artesunate onto poly (lactic-co-glycolic)acid nanoparticles by oil/water emulsion evaporation method [82]. In vitro cytotoxicity evaluation of the drug loaded nanoparticles on A549, SCC-7, and MCF-7 cancer cell lines showed that the nanoparticles had significant effect against the cancer cell lines. The formulations were very stable and effective as anticancer agent [82]. Some researchers reported the physicochemical properties of nanoparticles loaded with artemisinin and its derivatives which confirmed that they are potential therapeutic agents [89,90]. Chadha et al. complexed artesunate with β-cyclodextrin and loaded onto chitosan/lecithin nanoparticles with enhanced antimalarial activity [83]. The drug release behaviour from the nanoparticles was pH dependent and in vivo evaluation on mice infected with *Plasmodium berghei* by oral administration of the formulation showed that incorporation of artesunate onto the nanoparticle enhanced its therapeutic efficacy [83]. Sun et al. loaded dihydroartemisinin onto nanoparticles of gelatin and hyaluronan with anticancer activity [84]. In vitro analysis on A549 cancer cell lines showed that the nanoparticles loaded with drug, inhibited the cell growth more than the free drug. The anticancer effects of the formulation resulted from the aggregation of the drug with the nanoparticles in an electrostatic field environment [84]. Ibrahim et al. reported albumin nanoparticles loaded with artemisinin with antimalarial activity [85]. In vivo analysis on *P. falciparum*-infected humanized mice by intravenous administration revealed a 96% parasitemia inhibition effects of the nanoparticles loaded with drug at a dosage of 10 mg/kg/day [85]. Ma et al. developed poly(lactic-co-glycolic acid)-based nanoparticle and loaded with dihydroartemisinin and doxorubicin for combination chemotherapy [86]. Polymeric-nanoparticles therapeutic efficacy as a leishmanial agent in vivo [80], as anticancer agents [82,84] and as antimalarials [83,85] indicate that polymeric-nanoparticles are potential systems for the treatment of cancerous and protozoal infections. However, the size of the nanoparticles can result in particle-particle aggregation making their handling and storage difficult.

##### 4.8.2.2. Inorganic-Based Nanoparticles

Wang et al. reported the effect of magnetic nanoparticles of Fe_3_O_4_ on the anticancer activity of artesunate [87]. In vitro evaluation of K562 cell lines revealed a significant cell growth inhibition and apoptosis rate when cell lines were treated with nanoparticles together with co-polymer of artesunate more than the free drug [87]. Chen et al. loaded artemisinin onto iron nanoparticles with antitumor efficacy [88]. In vitro evaluation on HeLa cell lines revealed that the formulation inhibited cell growth. Incorporation of artemisinin derivatives onto the inorganic-based nanoparticles reduced their side effects and enhanced their biological activity. However, there are very few research reports and there is need for further studies.

## 5. Conclusions

Artemisinin and its derivatives have been reported to be effective as antitumor, anticancer, anti-inflammatory, antiviral and antibacterial agents. They are also effective for the treatment of other protozoan infections. Their poor bioavailability, poor water solubility and short half-life have motivated researchers to develop delivery systems that can improve their therapeutic efficacy. Some researchers developed and used known techniques to develop formulations encapsulated with artemisinin and its derivatives resulting in formulations with excellent bioavailability, stability, pharmacokinetic mechanisms and reduced toxicity than artemisinin and its derivatives.

In the application of delivery systems for the delivery of artemisinin and its derivatives, the design of the systems affects their therapeutic efficacy. In the design of polymer-drug conjugates for the delivery of artemisinin and derivatives, the nature of the targeting moieties affect the specificity of the conjugates, materials used influence their degree of toxicity, the type of drug linkers determine the drug release mechanism from the conjugates and the position of functionalities on the conjugates affect their rate of degradation. The design of micelles influence their degree of toxicity, rate of biodegradation and the rate of diffusion of the incorporated drugs. However, some of these delivery systems are limited by aggregation upon storage, burst drug release effects and susceptibility of selected drug linkers to some enzymes resulting in rapid drug release. There are few reports on the delivery of artemisinin and its derivatives from selected systems such as ethosomes, nanocapsules, niosomes and CNTs indicating that there is a need for further investigations on these systems. Most of the systems were evaluated in vitro and in vivo and the results obtained were promising suggesting that there is a pressing need for further studies to be performed on these systems to reach clinical trials because emergence of drug resistance remains a global problem.

## Figures and Tables

**Table 1:** Biological activity of reported hybrids of artemisinin.

Artemisinin Hybrid Compounds	Biological Activity	In Vivo/In Vitro Outcome	Ref.
Artesunate-indoloquinoline hybrid **6**	Antimalarial	The hybrid compound exhibited decreased cytotoxicity and increased antimalarial activity compared to the individual compounds. A dose of 10 mg·kg^−1^ once a day for four consecutive days resulted in a significant reduction of parasitemia.	[9]
Artesunate-podophyllotoxin analogue **7**	Anticancer	The hybrid compound exhibited good cytotoxicity effects on cancer cell lines with reduced resistant factor. It induced D G2/M cell cycle arrest in multidrug resistance K562/ADR cells.	[10]
Artesunate α-aminophosphonate analogue **8**	Antimicrobial	The hybrid compound exhibited significant activity against Staphylococcus aureus, Escherichia coli, Pseudomonas aeruginosa and Candida albicans microbials.	[11]
Artesunate-safranol analogue **9**	-	-	[12]
Primaquine-artemisinin analogue **10**	Antimalaria	The hybrids were more effective against the liver and blood stages of malarial parasites compared to the individual compounds.	[13]
Arteminisin derivatives containing Mannich base group	Antimalaria	The derivatives were very stable and effective against *Plasmodium berghei* in mice when compared to the free artesunic acid.	[14]
β-Ether derivatives of dihydroartemisinin	Antimalaria	-	[15]
Artemisinin derivatives **11** and **12**	Antimalaria	The derivatives exhibited good antimalarial activity.	[16]
Artemisinin-quinine hybrids **13**	Antimalaria	The hybrid compounds exhibited potent antimalarial activity against the 3D7 and drug-resistant FcB1 strains of *Plasmodium falciparum* in vitro than the individual drugs	[17]
Artemisinin-acridine hybrids	Antimalaria and anticancer	The hybrid exhibited seven-fold higher anti-gametocytocidal effect. The anticancer activity against the HeLa cells was between three to eight fold higher than the individual drugs.	[18]
Pyrrolidine-acridine-artemisinin hybrid	Antimalarial	The antimalarial activity of the hybrid was dose dependent resulting in haem bio-mineralization inhibition. It was effective for the treatment of multiple drug resistant with no sign of toxicity in vivo.	[19]
Artemisone and artemiside derivatives	Antimalarial	The derivatives increased drug concentrations in the combination therapy without reaching toxic levels and were active against *Plasmodium falciparum*.	[20]
Artemisone derivatives	Antitumor	In vitro activity of the derivatives on human hepatoma SMMC-7721 cell lines revealed that the derivatives inhibited proliferation of the liver cancer cells by inducing apoptosis.	[21,22]
Artemisinin derivatives	Anticancer	The derivatives were stable at room temperature, disrupting drug-resistance pathways when compared to artesunate.	[23,24]
Dihydroartemisinin-cinnamic acid hybrids **14**	Anticancer	In vitro antitumor activities of the hybrids against PC-3, SGC-7901, A549 and MDA-MB-435s cancer cell lines revealed that the hybrids were active against lung cancer.	[25]

**Table 2:** Polymer-drug conjugates and micelles containing artemisinin derivatives.

Polymer	Artemisinin Derivatives	Drug Delivery System	Application	Advantages	Ref.
Polyethylene glycol	Dihydroartemisinin	Polymer-drug conjugate	Antitumor	Enhanced solubility, long circulating half-life and improved antitumor effect.	[40,42]
Hydroxypropyl-β-cyclodextrin	Dihydroartemisinin	Polymer-drug conjugate	Oral administration	Increased solubility and stability of dihydroartemisinin.	[41]
Cyclodextrin via vinyl-acyl fatty esters	Artemisinin	Polymer-drug conjugate	Antimalarial by injection.	Effective antimalarial effect against *P. falciparum*.	[43]
Chitosan	Artemisinin	Polymer-drug conjugate	-	Improved solubility of artemisinin than the free artemisinin.	[44]
Polyorgano-phosphazenes	Primaquine and dihydroartemisinin	Polymer-drug conjugate	Antimalarial	Effective for combination therapy against drug resistant strain of *P. berghei* infected mice with 100% antimalarial activity.	[45]
Methoxy-polyethylene glycol	Artemether	Micelles	Antimalarial	Enhanced drug stability with prolonged release of artemether.	[46]
Polyurethane	Arteether	Micelles	Anticancer	Significant inhibition of the growth of 4T1 cell line.	[47]
PEG-PCL	Artemisinin	Micelles	Antitumor	High cellular uptake and inhibition effects on cancer cell lines with good antitumor efficacy.	[48]
Methoxy poly(ethylene glycol)/poly(l-lactic acid)	Dihydroartemisinin	Micelles	Anticancer	Better solubilizing ability than dihydroartemisinin suspension.	[49]

**Table 3:** Liposomes, nanocapsules, niosomes and ethosomes containing artemisinin derivatives.

Artemisinin Derivative	Drug Delivery System	Application	Advantage	Ref.
Artemether	Lipososmes	Anticancer	Enhanced sonodynamic anticancer activity.	[52]
Artemisinin	Liposomes	Anticancer	Enhanced cytotoxicity effects on MCF-7 cell lines.	[53]
Artemisinin	Liposomes	Antimalarial	Immediate and enhanced antimalarial activity.	[54]
Artemeter	Liposomes	Anticancer	Effective for invasive brain glioma.	[55]
Artemisinin	Liposomes	Antitumor and antiparasitic	Enhanced blood-circulation time and prolonged half-life.	[56]
Artemisinin	Magnetic liposomes	Anticancer	Thermosensitive with high antiproliferative activity.	[57,59]
Artesunate	Liposomes	Antitumor	Enhanced antitumor effects on human hepatoma HepG2 cells than the free artesunate	[58]
Dihydroartemisinin	Liposomes	Anticancer	Enhanced anticancer activity.	[60]
Artemisinin	Nanocapules	Anticancer	Prolonged release mechanism with enhanced bioavailability	[61,62,63,64]
Artemisone	Niosomes	Anticancer	Enhanced selective anticancer activity towards human melanoma A-375 cells with no toxicity towards the normal skin cells.	[65]
Artesunate	Niosomes	Anticancer	The release of artesunate from the formulations were slow and sustained with enhanced inhibitory effects against MCF-7 and C6 cell lines.	[66]
Artesunate	Ethosomes	Antimalarial	Enhanced permeation effect with antimalarial activity against *Plasmodium* parasite with no sign of re-emergence of malaria infection.	[67]

**Table 4:** Therapeutic outcomes of carbon-based, lipid-based, polymer-based and inorganic-based delivery systems loaded with artemisinin and derivatives.

Artemisinin Derivatives	Composition	Drug Delivery System	Application	Advantage	Ref.
Artemisinin	Multi-walled carbon nanotubes	Carbon-based	Anticancer	Enhanced inhibitory effect on K562 cancer cell lines.	[72]
Artemisinin	Multi-walled carbon nanotubes	Carbon-based	Anticancer	Enhanced antitumor effects in tumor-bearing murine model.	[73]
Artesunate	Fullerene, hyaluronic acid	Carbon-based	Anticancer	Excellent antitumor activity.	[74]
Artemisinin dimer piperazine derivatives	l-α-Phosphatidyl-choline extract from egg	Lipid-based nanoparticles	Anticancer	Enhanced inhibition of the growth of breast cancer cells and induced down-regulation of HER Family Members.	[76]
Areether	Soya lecithin, Tween 80 and Pluronic F68	Lipid-based nanoparticles	Antimalarial	Enhanced bioavailability.	[77]
Dihydroxyartemisinin	Miglyol^®^ 812	Lipid-based nanoparticles	Antimalarial	Enhanced bioavailability.	[78]
Artemether	Glyceryl trimyristate (solid lipid) and soybean oil	Lipid-based nanoarticles	Antimalarial	Improved bioavailability and biocompatibility.	[79]
Artemisinin	Poly lactic co-glycolic acid	Polymer-based nanoparticles	Antileishmanial	Significant reduction in the parasite load in the liver and spleen than the free artemisinin.	[80]
Artemisinin	Poly(ε-capro-lactone)	Polymer-based nanoparticles	Antimalarial	Sustained delivery of artemisinin.	[81]
Artesunate	Poly(lactic-co-glycolic)acid-	Polymer-based nanoparticles	Anticancer	Inhibitory effect of the nanoparticles loaded with drug was enhanced on A549, SCC-7, and MCF-7 cancer cell lines.	[82]
Artesunate	β-Cyclodextrin, chitosan/lecithin	Polymer-based nanoparticles	Antimalarial	Enhanced drug stability and antimalarial activity.	[83]
Dihydroartemisinin	Gelatin and hyaluronan	Polymer-based nanoparticles	Anticancer	Significant inhibition of the proliferation of A549 cells	[84]
Artemisinin	Albumin	Polymer-based nanoparticles	Antimalarial	Excellent bioavailability.	[85]
Dihydroartemisinin	Poly(lactic-co-glycolic)acid-	Polymer-based nanoparticles	Anticancer	Enhanced anticancer activity.	[86]
Artesunate	Fe_3_O_4_	Inorganic-based nanoparticles	Anticancer	Significant cell growth inhibition and apoptosis rate of K562 cell lines.	[87]
Artemisinin	Iron nanoparticles	Inorganic-based nanoparticles	Anticancer	Enhanced inhibition of HeLa cell lines.	[88]
